# What is the impact of patient recruitment on offshoring of clinical trials?

**DOI:** 10.1186/s40504-020-00104-4

**Published:** 2020-09-21

**Authors:** Maryam Kermanimojarad

**Affiliations:** Department of Public Policy and Social Change, Collegio Carlo Alberto, Turin, Italy

**Keywords:** Offshoring, Cost-related factor, The recruitment process, Clinical trails

## Abstract

The issue of globalization of research is receiving considerable attention due to the increasing number of offshored R&D activities from the United States, Europe, and Japan. This paper explores this phenomenon and provides a model to analyze the factors that will likely contribute to a global transformation of clinical trials. By identifying the main characteristics of clinical trials, I aim to clarify the main driver of the relocation process of clinical research. I reviewed the relevant published articles to address the research questions. The results of this study challenge the traditional thinking of cost-related factors as the major reason for offshoring cilinical trials and show the importance of the recruitment of human subjects in trials. Consequently, this paper suggests that “recruitment crisis” in home country as the main contribution and a key driver to offshore R&D activities, has been underestimated by previous studies. In particular, this study provides policy-decision makers with a new insight into the development issue surrounding the pharmaceutical industry.

## Introduction

With access to essential medicine being one of the building blocks of the healthcare system, policy measures aimed at reducing healthcare spending growth at the international level have targeted primarily the pharmaceutical industry, over the past decade (Settanni 2017). The pharmaceutical sector is one of the most important industries overall and presents the highest research and development (R&D) intensity in the US and EU. The United States Pharmaceutical R&D expenditure has grown from 2 million USD in 1980 to 79.6 million USD in the year 2018, according to a survey of PHARMA members. Clinical trials (CT), a major part of R&D in pharmaceutical companies, have received much attention, over recent decades. However, globalization has led to the extension of clinical research outside higher-income regions, followed by a growing trend towards the off-shore outsourcing of CT to “nontraditional locations’ such as Eastern Europe, China, and India (Cooper 2008; Clark and Newton 2004; Murthy et al. 2015). A considerable amount of literature has been published on cost-related factors for the internationalization of R&D activities, particularly as an explanatory factor for offshored CT. Nevertheless, several studies have already drowned attention to these widely held beliefs among academics and have risen the question that supply-related drivers (cost-related factors) have very limited explanatory power (Haeussler and Rake 2017).

This paper has been done in a narrative review and is descriptive and interpretative in nature but has used a systematic and developing process. The initial stage of any review method is a clear identification of the conceptual problem. In this regard, this paper has divided into two-part, the first part identifying the problem, and the second part, which provides an approximate answer to the research questions. The criteria for selecting the article in the first part of the paper is based on clinical trial characteristics. This provides both a theoretical framework and a specific analytical tool to help the author to address and select the articles that provide precise information on these characteristics and eliminate studies that are not aligned with it. The search for literature has targeted several categories of literature: peer-reviewed journals, conference papers, and Gary papers by employing search terms in various combinations such as “clinical trials and cost, clinical trials and time, clinical trials and succeed rate or probability of success”. The literature has found from these sources initially explored through their abstracts. By identifying the importance of Therapeutical areas in clinical research, in the next step, the author focuses on two countries, China and India. that the agent cost of the agent can not fully justify this issue, the author explains what the probable explanation of the OCT is.

This paper creates a solid starting point for clinical research in general and offering CT in particular. The purpose is to provide a novel synthesis of existing literature, which leads to new ways of looking at OCT and identifying gaps in the literature. In addition, this article introduces a well-defined framework that provides guidelines for the author to potentially not include thousands of publications, which would make the review unhelpful. One of the advantages in this paper is a step-by-step developing process based on a logic search strategy. Although there is a detailed and comprehensive search strategy that has developed, the risk of selection and publication bias remains.

In particular, this research will examine three main research questions:

1. What is the main driver of the offshored outside of the US?
2. Are there differences among the therapeutical areas that have been offshored?
3. What are the motivations behind choosing the location of offshored CT?

The overall structure of the study takes the form of four parts, including this introductory part; chapter two begins by presenting the specific characteristics of CT, plus defines how these characteristics impact on the value of each phase that they add to the research activities. Chapter three analyses the offshoring of CT. Finally, the conclusion gives a summary and critique of the analytical part and will close with some essential questions related to policy-decision making.

## Main text

### The main characteristics of clinical trials

The research and development (R&D) in the pharmaceutical industry starts with discover research that includes basic research, target discovery & validation and drug design, aiming at collecting early evidence whether the drug candidate acts in humans. Next phase is preclinical research drugs which undergo laboratory and animal testing to answer basic questions about safety (Balconi and Lorenzi 2017). After successfully completing preclinical studies, scientists file an investigational new drug application with the FDA, outlining the preclinical study results and a detailed plan for the clinical study program in humans. These studies- known as CT- are designed to demonstrate the medicine’s safety and efficacy and comprise four major phases:

Phase I is usually conducted with healthy volunteers to assess the safety of a drug candidate and medicine is tested in a small group (100 or less). Phase II tests drug candidate in a somewhat larger group (100 to 500) of patient volunteers living with the disease. Phase III trials employ larger sample sizes (usually in the thousands) to evaluate the safety and efficacy within different populations and by using different dosages to determine the overall benefit-risk ratio. If CT results show the compound is safe and effective, the sponsoring company submits a New Drug Application or a Biologics License Application to FDA seeking review and approval to start with the marketing. At the end, after the FDA approves the new drug, company scientists work to identify the best way to manufacture and packing the new medicines for patients. Research on a new medicine doesn’t stop when it receives FDA approval. Phase IV studies are conducted after market approval to gather additional information concerning a drug’s safety, efficacy, and optimal use (PhRMA 2016; Haeussler and Rake 2017; Azoulay 2004).

Beyond any doubt, all new medicines introduced into the market are the result of lengthy, costly and risky research and development conducted by pharmaceutical companies (EFPIA 2018; PhRMA 2016).

Regarding to cost of reserch activities, studies estimate that it costs somewhere between US$161 million and US$2 billion to bring a new drug to market. The average cost of developing a drug had risen at a rate of 7.4% higher than inflation over the past two decades, mostly due to rising CT costs. Costs also tend to increase as an investigational drug progresses through each phase of the R&D process, and, as the Institute of Medicine notes, Phase III CT have become “extraordinarily expensive” (Sertkaya et al. 2016). According to a report by PhRMA (2016), the allocation of R&D investment in a CT is around 49% of the overall costs, and Phase III alone constitutes about 29% (the same results reported by EFPIA 2018).

**Fig. 1:**
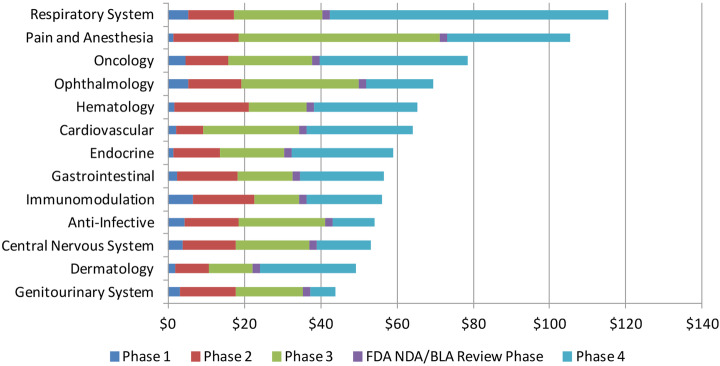
The average per-study costs for each of the therapeutic areas by phase. Source: Sertkaya et al. (2014) & Sertkaya et al. (2016)

However, it should be taken into account that the average cost of conducting CT is different across therapeutical areas (Fig. 1).

As Sertkaya et al. 2016, reveals, while Phase III tends to be extraordinarily expensive, clear differentiation is emerging when accounting the average costs for each phase across therapeutical areas. For example, the average cost of genitourinary system for whole phases is around 42$ million, while the average cost in anesthesia for Phase III only is around 60$ million. In addition, the average cost for phase II in hematology is higher than the average cost of phase III in dermatology. Thus, some therapeutical area presents higher cost for pharmaceutical companies, that have to spend more money on conducting them in comparison with other areas.

In terms of length of R&D process, conducting CT often requires a remarkable time, another challenging aspect for the research-based pharmaceutical industry. By the time a medical product reaches the market, an average of 10–15 years will have elapsed since the first synthesis of the new active substance (EFPIA 2018). This average, however, masks substantial differences among different therapeutical areas (Kaitin 2010).

In a survey, Wong et al. (2019) provide an estimation for the duration of CT using a sample of 406.038 entries of CTdatas from 2000 to 2015, finding that the median CT durations are 1.6, 2.9, and 3.8 years, for trials in Phases I, II, and III, respectively. The findings for Phase III are higher than others, while for Phase I is lower. In the next step, they examine the individual durations across Phases I through III and across the therapeutical areas, and find that the median time spent in the clinic ranged from 5.9 to 7.2 years for non-oncology trials, while the median duration for oncology trials was 13.1 years. It appears from previous study that the most surprising aspect of the datas is in the Oncology and this suggests higher risks in oncology projects and may explain their lower approval rate. Therefore, any drug approval in this therapeutical area should bring huge revenues for the pharmaceutical companies. As a result, it is become obvious that therapeutical areas are a determinant key in terms of length (IQVIA 2019).

Another important component of R&D value chain in the pharmaceutical industry is related to the Success Rate and Probabilities of Clinical Phase Transition. The probability of success (POS) of a CT is critical for clinical researchers and pharma investors to evaluate when making scientific and economic decisions (Wong et al. 2019). According to the EFPIA report (2018), on average only one to two of every 10,000 substances synthesized in laboratories will successfully pass all stages of development required to become a marketable medicine. In another report from PhRMA (2016), only 12% of candidate medicines that enter CT are actually approved. Wong et al. (2016) claim that Phase II trials have the lowest tendency to complete, while Phase III trials, that are often larger-scale replications of Phase II trials, and thus potentially riskier and costlier, yet they have a higher completion rate than Phase II trials. Possible explanations include selection bias and commitment, as only the most promising trials in Phase II are selected for Phase III trials, since phase III is tending to be costlier.

Nevertheless, it should be taken into account that the success rate of drug development varies among different therapeutical areas. Wong et al. (2019) show that the overall POSs across the different therapeutical groups move in tandem over time. There are some minor deviations, such as the POS of drugs and vaccines for infectious diseases increasing between 2005 and 2007. Nonetheless, the results suggest that there is a systemic factor driving the trends over time. The important point is that Oncology trials performed much more poorly than average of all trials concluding successfully. A closer look shows that their completion rates were lower across all phases (Kaitin 2010; Wong et al. 2019). In the years from 2009 to 2018, the composite success rate for oncology products averaged 12.0% compared to 14.1% for all other products (IQVIA Institute 2019).

This overall picture across the unique characteristics of R&D reveals that while these characteristics, including time, cost and success rate are important in the R&D, we should not neglect the therapeutical areas that provide a specific tool for assessing each phases of CT. In addition, the therapeutical class also impact on the intellectual property rights through the patent system. It is said that introducing new drugs produces an enormous amount of value for pharmaceutical companies. Those benefits from new drugs stem in great measure from patent policy and the granting of marketing exclusivity to this new drug products. This means that if free competition were permitted, firms spending hundreds of millions of dollars to bring a new drug to market would be unlikely to recoup those investments. Therefore, it is these features of the economics of new drug development that make the establishment of intellectual property rights through the patent system and regulation of marketing exclusivity so important to promoting R&D investment profitable. However, the fact that patents are granted and marketing exclusivity for new drugs is established does not mean there is no competition. Competition between patented drugs that treat the same medical conditions does occur, but it is based on the therapeutical area of the drugs and to a more limited extent on price. This is referred to as “differentiated” product competition. In the case of differentiated competition, pharmaceutical companies will tend to pursue R&D investments where the size of markets and the potential price-cost margins are greatest. For example, prevalent cancers, in the hope of realizing large returns (Frank and Ginsburg 2017). This is why this therapeutical class, with a high length of conducting CT, low level of success rate and to some extent costly trials, represents a high appealing area for pharmaceutical companies to invest in. According to IQVIA Institute (2019), the number of CT initiated in 2018 is up 9% over 2017, due partly to an increase in Phase II oncology trials.

In another hand, during planning a trial, one essential step is the calculation of a sample size that will give the minimum number of participants required to meet the objectives of the study (Julious 2010). Poor recruitment is acknowledged as an important shortcoming of many randomized controlled trials, which can prevent a study from reaching its target sample size. A commonly reported problem with the conduct of multicentre randomized controlled trials (RCTs) is that recruitment is often slower or more difficult than expected, with many trials failing to reach their planned sample size within the timescale and funding originally envisaged (McDonald et al. 2006). Of trials published in the British Medical Journal (BMJ) and the Lancet between 2000 and 2001, 51% of multicentered trials reported difficulties in recruitment (Sully et al. 2013).

Kitterman et al. (2011) report the results of an evaluation of the prevalence and cost of low-enrolling studies (zero or one participant enrolled) conducted at Oregon Health & Science University (OHSU). They found that one-third of all studies terminated between 2005 and 2009 at OHSU had low enrollment and that these low-enrolling studies cost the institution almost $1 million annually.

The recruitment of research participants is critical to conducting clinical and translational research. Failure in recruiting research participants has a negative financial impact, but, more importantly, under-enrolled studies do not contribute to scientific or clinical knowledge (Nasser et al. 2011). A large number of trials are dependent on the willingness of patients and professionals to give their time and effort to participate. If high levels of participation (through recruitment to the study and longer-term retention) are not achieved, this has implications for statistical power, internal validity, and external validity. Recruitment problems also have practical impacts, as they can delay completion of research or reduce its timely impact on patient health and wellbeing (Bower et al. 2014). Participation in the CT has varied greatly by phases and across therapeutical areas, with some trials having fewer than ten patients to others including several thousand (FDA 2018). In addition, gender, age and race also play an important role in the patient recruitment issue. For instance, CT in ovarian cancer require the participation of only women, not men. As a result, the nature of therapeutical areas also limits the number of eligible participants in CT.

To sum up, Clinical trials involve the human. One should consider the patient as an inevitable element of the R&D process. Therefore, the recruitment of patients is known to be one of the most challenging aspects in the conduct of CT. Inadequate patient retention during the conduct of trial affects conclusive results. Recruitment and retention issues can adversely affect the ability to detect intervention effects and may limit the significance of the research findings (Chhatre et al. 2018; Bower et al. 2014). Therefore, returning to the characteristics of CT, it is now necessary to consider the patient recruitment issue in CT.

Consequently, it can be seen that the R&D process is surrounded by two important components: therapeutical area and recruitment process. Both putting some barriers for pharmaceutical companies. They are interrelated and both impact on assessing the caracteristics of CT. It is also worth noting that the patient is a receiver and a provider of services in CT. It is impossible to imagine any trials without the patient. Concerning these important components, I will try to address the offshoring phenomenon in CT and answer the research questions.

### Offshoring of clinial trials

Over the last decade, the extent and character of trends towards the internationalization and globalization of research activities have been subjects of lively debate. However, the economic consequences of this phenomena are still not clear and have been controversially debated (Haeussler and Rake 2017; Lang and Siribaddana 2012). Several studies have been made to examine the motivations behind the international location of offshored R&D activities and address the supply and demand-side factors. Supply-side factors have been identified as major contributing factors for internationalization of R&D. In this regard, the cost-effectiveness and efficiency gains seen as the main endeavor (Dezfuli 2017; Agalew 2013; Mackie and Oss 2008). Mirowski and Van Horn (2005) argue that pharmaceutical companies benefit from lag in regulatory oversight. In addition, in the developing countries ethical enforcement is likely to be looser than in the developed countries, which also raised the concern over ethical variability in global CT (Rajan 2007).

According to Pharma Intelligence, CT activity in China has been on the rise from 2007 to 2016, although the data shows the waning of trial volume from US and European Big Pharma after 2010, while Chinese companies started to emerge as new leading sponsors. The majority of industry sponsors of China’s trials in 2007 were large US pharma companies (about 5 of 10 top companies) and a few European companies. When CT in China are grouped by major therapeutical areas (TA), their activity transformed from dabbling across six TAs to a heavy focus in oncology, with only a handful of trials in two other CT as (include Cardiovascular and Metabolic/Endocrinology) (Chen 2017). In an analysis of CT in India, Mondal and Abrol (2015) show that India hosts 5% of global CTand out of these, 60% are Phase III and IV trials, while only 5% are Phase I trials. India accounts for 21% of the global burden of disease in two major groups: The burden of “communicable” diseases - TB, Malaria, HIV, water-borne and vector-borne diseases - in the country is very high, especially among children and mothers, which poses serious health problems. Similarly, the burden of “non-communicable” diseases like cancers, diabetes, mental health disorders, and cardiovascular disease (CVD) is also high and is the leading cause of functional impairment and death. CTRI data shows that between 2007 and 2009, out of 116 foreign companies, 110 concentrated on non-communicable diseases include cancers, diabetes and cardiovascular disease (Abrol et al. 2011). In another research, Chaturvedi et al. (2017) indicate that the top five areas in which CT were conducted included cancers, diabetes mellitus, cardiovascular diseases, musculoskeletal diseases, and digestive diseases between 2007 to 2010 while the top five health conditions contributing to the disease burden were infectious and parasitic disease, cardiovascular diseases, neonatal conditions, respiratory diseases, and mental and behavioral diseases.

**Fig. 2:**
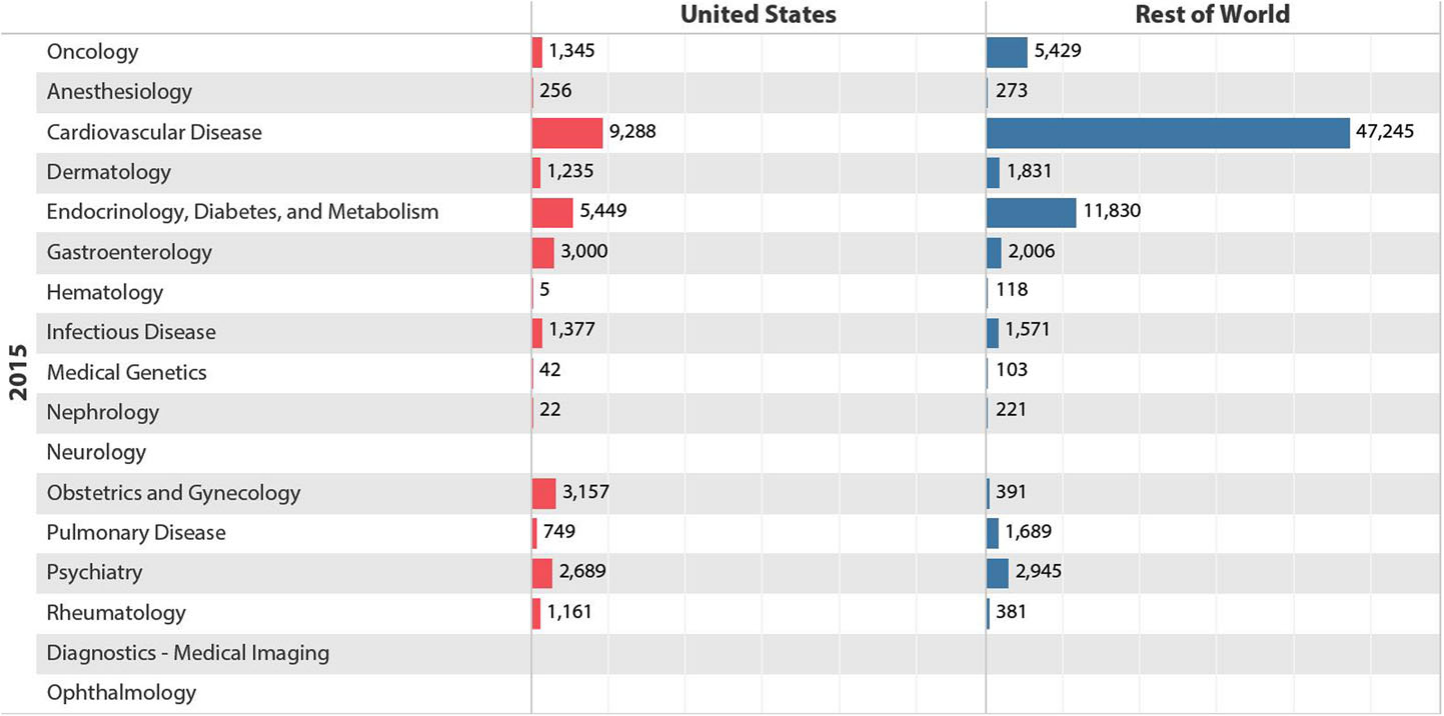
Trial Participants by Therapeutic Area, and Geography. Source: FDA report 2017

Previous studies reveal a specified trend in offshored clinical trials that have been focused mostly on some certain therapeutical areas including cardiovascular, metabolic and oncology. A report by FDA (2017), show the proportion of the CT on oncology, cardiovascular, and metabolism that have been conducted in 2015 within the US were less than the proportion of these areas in the rest of the world, while in other therapeutical classes the proportion of CT were the same or more than in the rest of the world (Fig. 2).

**Fig. 3:**
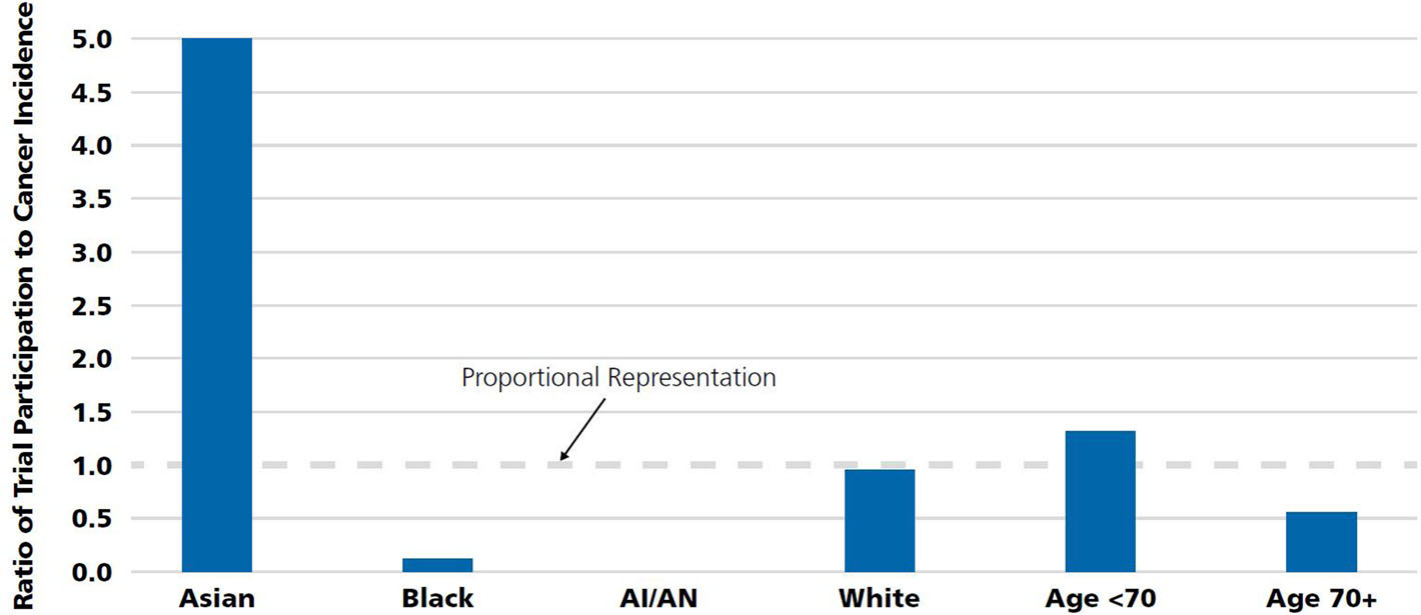
Demographic representation in FDA-submitted cancer trials. Source: American Cancer Society Cancer Action Network (ACS CAN) (2018)

A recent study by Kanapuru et al. (2017) quantifies and characterizes participants in oncology trials from 2005 to 2015 in the US. He found that 45 % (80.460) of CT participants were enrolled from Europe, 36 % (63.958) from North America (includes U.S.A and Canada) and 8.4% (14.975) from Asia. Countries in Latin America, Middle East/Africa and the Baltic States/Russia enrolled the remaining 10.5% of the patients. Among 99.556 participants < 65 years of age, 38.7% (38.538) were enrolled from North America, 40.5% (40.362) from Europe, 9.7% (9.674) from Asia and 11% from the rest of the regions. Europe enrolled the highest number of cancer patients aged 65 years or older; 51.1% (40.098) compared to 32.4% (25.420) from North America and 6.8% (5.301) from Asia. Consequently, the majority of patients enrolled in CT submitted for oncology drug approvals were from regions other than North America, with the highest number enrolled from Europe. It is interesting to speculate the reasons for differential enrollment of patients between Europe and North America and other countries. In another major study, Singh et al. (2017) examined demographic data of cancer patients enrolled onto trials between 2005 and 2015 according to age distributions between 65 and 80 years old. They found over-representation of Asians and significant under-representation of Blacks and American Indians/Alaska Natives (AI/AN) in data (Fig. 3).

This study reveals that not only some therapy area has been offshored but offshoring CT have been largely focused on a specific age group, mostly older people. In the survey by Haeussler and Rake (2017) reveal that no cost arguments are influencing the number of CT in developing countries between 2002 and 2012. Therefore, if there is no evidence of cost-benefit drivers for offshored CT, there should be another driver that motivates the pharmaceutical companies for conducting CT outside the US. In 2000, a curious metric on investigative site performance was presented at a small conference in Philadelphia and it quickly invaded the industry’s collective conscience becoming a widely cited “fact.” That metric, based on interviews among clinical operations managers, held that 20% of all investigative sites fail to enroll a single patient; 30% under-enroll; 30% of investigative sites meet enrollment targets; and 20% exceed target enrollment levels (Getz 2012). This survey shows that at least half of CT had not met their needs related to the required patient number. Dickert and his colleagues (Dickert et al. 2013) look at cardiovascular clinical trails and indicate that pervasive problems related to the recruitment and retention of participants in clinical research threaten clinical trails’ ability to produce timely data necessary to guide practice and policy. For example, the Warfarin Versus Aspirin in Reduced Cardiac Ejection Fraction trial took more than 7 years to recruit, averaging 1 patient per site about every 6.25 months. The Efficacy of Vasopressin Antagonism in Heart Failure: Outcome Study with Tolvaptan (EVEREST) trial involved 436 sites to enroll 4133 patients; 77 sites enrolled no patients, and the median enrollment among active sites was only 6. Related to this, a major barrier to both Food and Drug Administration approval and acceptance among cardiologists seems to have been uncertainty related to high rates of loss to follow-up among enrolled subjects. Although, these problems are not universal. The Acute Study of Clinical Effectiveness of Nesiritidein Decompensated Heart Failure study, for example, successfully randomized 7141 patients in just less than 3 years. Moreover, Unger et al. (2019) claim that the vast majority of adult patients with cancer do not participate in CT. It is commonly assumed that only 2%–3% of adult cancer patients participate in CT, even though most Americans view CT participation favorably. This finding approves the survey by Singh et al. (2017) that founds over-representation of Asian adults in cancer CT registered in the US’s FDA between 2005 and 2015.

It seems more challenging to recruit CTsubjects in certain therapeutical areas than in others. Several studies find that most of the cancer and cardiovascular CT don’t meet their enrollment targets due to institutional barriers or patient reluctance (Unger et al. 2019; Getz 2012). In the case of China and India, offshored CT(OCT) has been placed mainly in Phase III trials. The Phase III needs thousands of patients for conducting the trials and tends to be more difficulty in recruiting the patient. As Drain et al. (2018) points out, the overall global migration of operational CT sites has been done particularly for Phase III trials.

There are several possible explanations for the OCT, but a systematic approach used in the present study reveals “Recruitment Crisis” in home country as the major factor, if not the only one, causing the pharmaceutical companies to relocate their clinical research operations to India and China, this occurring because of the “larger patient pool” in these countries (Mondal and Abrol 2015). While the previous articles mainly address the issue of OCT in a broad perspective without identifying the type of therapeutical classes that have offshored and less pay attention to this aspect of CT.

Consequently, offshoring has been focused on the therapeutical areas that have to tackle the lack of availability of sufficient patients. OCT could be seen as a wise strategy taken by the pharmaceutical companies in order to pursue their goals concerning the R&D process. This is an important issue for future research, and future studies, which take these variables into account, will need to be undertaken. Especially in the case of the biopharmaceutical industry, the issue of recruitment crises will remain in the future of the health system. If patients do not wish to participate in CT or lack of recruitment, Clinical trials con not be conducted in other counties due to the nature of biomarkers and genomics issues in biotechnology.

## Conclusions

The present study is designed to address the main driver of offshored Clinical trials (OCT). One of the most significant findings emerging from this study is that “Recruitment Crisis” in home country could be the main explanation for OCT. This finding challenges the existing view and widely accepted belief regarding the relevance of cost-related factors for offshored outsourced R&D activities and has important implications for policy issue in the pharmaceutical industry. In fact, considering the cost-related factor as the main driver of OCT, first has defined offshoring as declining the number of trials in the home country and causing a smaller job market for who have education, knowledge and skills in that field. Second, it has convinced the policy-decision makers to prevent OCT and deprive the pharmaceutical companies from a wide and various patient pool across the world in order to protect the employment rate and national resources. However, offshoring allows pharmaceutical companies to increase the number of CTand a concurrent 20% improvement in productivity (Jones and Minor 2010).

Taken together, this study raises the question that cost reduction could be as a result of offshored clinical trials not as a driver. Conducting CT in India and China lead to the decreasing of trials costs regarding to the lower cost of labour in those countries compared to the home countries.^1^ This co-occurrence may have convinced the researchers to consider the clinical cost-minimization as the main reason for OCT.

This study makes several contributions to the current literature. First, patient both as a provider and a receiver of services for/from CT should be considered as a main component to the R&D process. Second, therapeutical class *plays a significant role* in conducting CTs and estimating the main characteristics of CT, including length, cost and success rate. Finally, this study put stress on the importance of “Recruitment Crisis” in home country for the policy decision-makers who concern the geography of CT. However, the major limitation of this study is the lack of sufficient data in OCT and this could have a negative impact on analyzing the drivers of OCT. This research has thrown up many questions in need of future investigation. Although the three important factors driving geographical shift are economic drive, the population for recruitment, and regulatory constraints, this paper takes more attention to the issue of recruitment as the main driver. It is recommended that the association of the regulatory constraints and ethical issues with therapeutic classes is investigated in future research to determine whether there is a discrepancy between the regulatory process among therapeutic areas and whether this could be an explanatory factor for OCT.

## Data Availability

Not applicable.

## Abbreviations

CT: Clinical Trials
OCT: Offshoring Clinical Trials
POS: The probability of success
ACSCAN: American Cancer Society Cancer Action Network
FDA: Food and Drug Administration
EFPIA: The European Federation of Pharmaceutical Industries and Associations
PhRMA: The Pharmaceutical Research and Manufacturers of America

## Footnotes

1 It has been seen that if the overall CTcost in USA is 1unit, then in India it would as low as 0.11 units (A.T. Kearney: Wang, 2005).
